# IL-10, IL-13, Eotaxin and IL-10/IL-6 ratio distinguish breast implant-associated anaplastic large-cell lymphoma from all types of benign late seromas

**DOI:** 10.1007/s00262-020-02778-3

**Published:** 2020-11-04

**Authors:** Arianna Di Napoli, Daniele Greco, Giorgia Scafetta, Francesca Ascenzi, Alessandro Gulino, Luigi Aurisicchio, Fabio Santanelli Di Pompeo, Adriana Bonifacino, Enrico Giarnieri, John Morgan, Rita Mancini, Marshall E. Kadin

**Affiliations:** 1 Department of Clinical and Molecular Medicine, Sapienza University of Rome, Sant’Andrea Hospital, Via di Grottarossa 1035, 00189 Roma, Italy; 2 Tumor Immunology Unit, Human Pathology Section, Department of Health Science, Palermo University School of Medicine, Palermo, Italy; 3 Department of Clinical and Molecular Medicine, Risk Management Q and A, Sant’Andrea Hospital, “Sapienza” University, Rome, Italy; 4 Takis Biotech, Rome, Italy; 5 Plastic Surgery Unit, Sant’Andrea Hospital, Sapienza University, Roma, Italy; 6 Breast Unit, Sant’Andrea Hospital, Sapienza University, Roma, Italy; 7 Department of Clinical and Molecular Medicine, Sapienza University, Cytology Unit, Sant’Andrea Hospital, Roma, Italy; 8 Department of Pathology and Laboratory Medicine, Albert School of Medicine, Brown University, Providence, Rhode Island USA

**Keywords:** BI-ALCL, Cytokines, Seroma, Diagnosis

## Abstract

Breast implant-associated anaplastic large-cell lymphoma (BI-ALCL) is an uncommon peripheral T cell lymphoma usually presenting as a delayed peri-implant effusion. Chronic inflammation elicited by the implant has been implicated in its pathogenesis. Infection or implant rupture may also be responsible for late seromas. Cytomorphological examination coupled with CD30 immunostaining and eventual T-cell clonality assessment are essential for BI-ALCL diagnosis. However, some benign effusions may also contain an oligo/monoclonal expansion of CD30 + cells that can make the diagnosis challenging. Since cytokines are key mediators of inflammation, we applied a multiplexed immuno-based assay to BI-ALCL seromas and to different types of reactive seromas to look for a potential diagnostic BI-ALCL-associated cytokine profile. We found that BI-ALCL is characterized by a Th2-type cytokine milieu associated with significant high levels of IL-10, IL-13 and Eotaxin which discriminate BI-ALCL from all types of reactive seroma. Moreover, we found a cutoff of IL10/IL-6 ratio of 0.104 is associated with specificity of 100% and sensitivity of 83% in recognizing BI-ALCL effusions. This study identifies promising biomarkers for initial screening of late seromas that can facilitate early diagnosis of BI-ALCL.

**Electronic supplementary material:**

The online version of this article (10.1007/s00262-020-02778-3) contains supplementary material, which is available to authorized users.

## Introduction

Breast implant-associated anaplastic large-cell lymphoma (BI-ALCL) is a provisional entity recently introduced in the revised version of the WHO classification of lymphoid malignancy [1]. Most patients experience a late-onset peri-prosthetic effusion (seroma) as the first manifestation of the disease [2]. Aspirated effusions must undergo microbiological culture, cytomorphological examination, immunocytochemistry for CD30 expression and sometimes analysis of T-cell receptor genes rearrangement to confirm clonality of the T-cell population. This approach allows an early diagnosis and avoids local and lymph node metastasis of tumor cells [3, 4]. Nevertheless, most late seromas are benign and related to infection, trauma or implant rupture [5]. Moreover, selected cases presenting with a suspicious clinical history, negative culture and cytology and positive molecular test may be difficult to classify [5, 6]. Indeed, some reactive inflammatory responses may be associated with a T-cell oligo-monoclonal expansion that may make the diagnosis even more challenging. Hanson et al. reported on a CD30 enzyme-linked immunoabsorbant assay for the rapid detection of breast implant-associated anaplastic large-cell lymphoma [7]. However, a major limitation to the assay is represented by CD30 + reactive T cells being potentially detected in lymphocyte-rich benign seromas [5, 6]. Recently, higher levels of soluble interleukin (IL) 9, IL-10, IL-13, IL-22, and/or IFN-γ have been detected in malignant seromas than in benign seromas [8]. This prompted us to further validate these results and to investigate the existence of a possible BI-ALCL-associated cytokine signature by applying a highly multiplex immuno-based assay to late seromas, including, besides BI-ALCL, different types of reactive effusions.

## Methods

### Samples

Late peri-implant breast seromas aspirated by ultrasound-guided fine-needle aspiration (US-FNA) and sent for cytological analysis were centrifuged and supernatants were collected and stored at -80 °C. Cell pellets were immediately used for smear and cell block preparation. Based on morphological and immunohistochemical analyses, the seromas were diagnosed as malignant (12 BI-ALCL samples) or benign (20 samples). The latter were further sub-classified as acute-type (6 samples), mixed-type (6 samples) and chronic-type (8 samples) as previously reported [5]. All patients with BI-ALCL were women and had textured implants placed either for reconstructive (6 patients) or cosmetic indication (6 patients); their ages ranged from 35 to 76 years (mean, 55 years), and time from implantation to seroma development ranged from 4 to 15 years (mean, 8.6 years). Also, evaluated were supernatants of 7 T-cell lymphoma cell lines (T-LCL), derived from clinical cases of BI-ALCL (TLBR-1,-2,-3,-4) [9], cutaneous T cell lymphoma lines (Mac-1, Mac-2A established by MEK) and an ALK-positive systemic ALCL (Karpas 299) obtained from DSMZ German Collection of Microorganisms and cell cultures GmbH.

### Cytokine detection

The Human ProcartaPlex Cytokine/Chemokine/Growth Factor 45-plex immunoassay kit (Invitrogen by Thermo Fisher Scientific) was adopted to simultaneously analyze the concentrations of 45 different cytokines in the supernatant of 32 late seromas and of 7 cell lines following the manufacturer’s recommendations, in a Luminex Instrument system. Samples were run in duplicate as undiluted and diluted to 1:20 in phosphate-buffered saline (PBS) and compared to a standard curve. The 45 cytokines and growth factors included: brain-derived neurotrophic factor (BDNF), epidermal growth factor (EGF), Eotaxin, fibroblast growth factor (FGF2), granulocyte–macrophage colony stimulating factor (GM-CSF), growth-related oncogene protein-α (GRO-α), hepatocyte growth factor (HGF), interferon-α (IFN-α), IFN-γ, interleukin 1 receptor antagonist (IL-1RA), IL-1α, IL-1β, IL-2, IL-4, IL-5, IL-6, IL-7, IL-8, IL-9, IL-10, IL-12p70, IL-13, IL-15, IL-17A, IL-18, IL-21, IL-22, IL-23, IL-27, IL-31, IFN-γ-inducible protein 10 (IP-10, CXCL10), leukemia inhibitory factor (LIF), monocyte chemotactic protein 1 (MCP-1), macrophage inflammatory protein type 1β (MIP-1β, CCL4), MIP-1α, NGF-β, chemokine (C–C motif) ligand 5 (CCL5, RANTES), platelet-derived growth factor-BB (PDGF-BB), placenta growth factor 1 (PIGF-1), stem cell factor (SCF), stromal cell-derived factor 1 (SDF-1α), tumor necrosis factor-α (TNF-α), TNF-β, vascular endothelial growth factor-A (VEGF-A), and VEGF-D.

### Data analysis

The expression levels of the 45 different cytokines assessed were compared among the samples using the averaged median fluorescent intensity (MFI) values obtained by the average of undiluted and diluted samples. Hierarchical clustering analysis (HCA) was performed using Spearman correlation distance and Ward.D2 linkage criterion [10] (implemented in the `hclust` function (R package `stats`). Dendrogram and heatmap were generated using the `ComplexHeatmap` package (version 2.2.0) (R package `stats`) [11]. To investigate misplaced samples with respect to clinical classification, multidimensional scaling (MDS) was performed using the `cmdscale` function (R package `stats`) retaining only the first three principal coordinates (explained variance: 70.4%) and a 3D plot generated using the `plot3D` (version 1.3) (R package `stats`) (https://cran.r-project.org/package=plot3D).

Differential analysis of cytokine concentration was conducted on the log2 transformed MFI values using a moderated *t*-test [12] implemented in the `limma` R package (version 3.42.2, Bioconductor version: release 3.1) [13]; *p *values were corrected for multiple testing applying the Benjamini–Hochberg procedure [14]; a leave-one out strategy was implemented to assess the robustness of differentially expressed genes in a given group comparison by counting the number of times a cytokine is differentially expressed (*p* < 0.01) removing any one sample from the dataset. Volcano plots, representing the distribution of the fold changes (difference of means, log2 scale, *x*-axis) and *p*-values (BH-adjusted, -log10 scale, *y*-axis) for each comparison, were generated using the `plot` function (R package `graphics`).

Group comparisons based on the mean absolute concentration levels for selected cytokines IL-10, Eotaxin, IL-13, IL-6, and IL-10/IL-6 ratio were again assessed by the means of moderated *t*-test, using the `limma` R package.

The ability of IL-10, Eotaxin, IL-13 and IL-6 cytokine levels (pg/mL) and of IL-10/IL-6 ratio to identify BI-ALCL samples was evaluated with receiver-operator characteristic (ROC) curves, including the computation of sensitivity, specificity, optimal cut-off values (Youden index), area under the curve (AUC), 95% confidence interval (CI), using the `pROC` (version 1.16.2) R package [15].

All statistical analyses were performed in R version 3.6.2 (https://www.r-project.org), using RStudio IDE version 1.2.1322 (https://www.rstudio.com/).

### Establishment of primary cell culture of BI-ALCL and xenotransplantation into NGS mice

Primary tumor cells derived from seromas of three patients diagnosed with BI-ALCL at Sant’Andrea Hospital, Roma, Italy were maintained in suspension culture in a complete medium (RPMI-1640 with 10% fetal bovine serum, 100 U/mL penicillin and 100 ug/mL streptomycin) supplemented with 50 IU/mL of recombinant IL-2 (R&D Systems). After two weeks, 2.5 × 10^6^ viable primary tumor cells derived from the 3 patients, were injected subcutaneously in the right flank of three six-week-old female NOD.Cg-Prkdcscid Il2rgtm1Wjl/SzJ mice (NSG; Charles River). Before injection, cells were washed once in PBS and the cell pellet was resuspended in Matrigel solution (SIGMA). NGS mice are severely immunodeficient due to the lack of mature B, T, NK cells and IL-2 signaling. After one week of acclimation, mice were housed in a plastic cage and fed on standard diet with water ad libitum, in an animal facility controlled at a temperature of 23 ± 2 °C, 60 ± 5% humidity, and with a 12 h light and dark cycle.

One of these primary cell lines was tumorigenic in the NGS mouse and after 83 days from injection, a discrete tumor mass was palpable. Tumor growth was monitored weekly by caliper measurement and tumor volume was determined by the formula (D × d^2^)/2, where D was the longest diameter of the tumor. The animal was euthanized by cervical dislocation when severe signs of suffering were observed. At sacrifice, a complete necropsy procedure was performed. No tumor cells were found in lymph nodes, lungs, liver or spleen. The subcutaneous tumor mass measured 8.49 mm × 7.87 mm (volume = 263 mm^3^), its weight was 160.4 mg and after removal, it underwent histopathological examination.

### RNAscope validation of selected cytokines

Cytokine mRNA production of IL6 and IL10 was further investigated by RNA in situ hybridization (ISH) using RNAscope technology. The RNAscope assay was applied to cell block paraffin sections of late seromas as previously described [16, 17]. Briefly, FFPE Sects. 2 μm thick were deparaffinized in xylene and then hydrated in an ethanol series. Hybridization was with target probes: Probe-Hs-IL6 and Probe-Hs-IL10. The preamplifier, amplifier, label probe, and chromogenic detection procedures were performed according to the manufacturer’s instructions (RNAscope® 2.0 HD Reagent Kit, Advanced Cell Diagnostics, Newark CA, USA).

For double staining, RNAscope assay for IL6 and IL10 was performed first and followed by immunohistochemistry for CD30 (clone Ber-H2, diluition 1:50) (Dako, Denmark). Staining was revealed using Super Sensitive Link Label IHC Detection System Alkaline Phosphatase (BioGenex, Fremont, CA, USA). Vulcan Fast Red Chromogen Kit 2 (BioCare Medical, Pacheco, CA, USA) was used as substrate-chromogens, followed by counterstaining with Harris hematoxylin.

### Immunohistochemistry

Immunohistochemistry for GATA3 (clone L50-823, Cell Marque, Rocklin, CA) and FoxP3 (clone 236A/E7, Abcam, UK) was performed on 2 µm thick formalin-fixed paraffin-embedded (FFPE) tissue sections of a BI-ALCL seroma cell block and of a BI-ALCL xenograft using an automated immunostainer (Omnis, Agilent Technologies, USA). Tissue sections were pretreated using EnVisionTM FLEX Target Retrieval Solution (Agilent) and incubated with an optimal dilution of the primary antibody. The reaction was visualized with the EnVision Detection Kit (Agilent) using 3–3′-diaminobenzidine chromogenic substrate. Sections were counterstained with EnVision FLEX Hematoxylin (Link) (Agilent).

## Results

### BI-ALCL has a distinct cytokine profile with significantly higher levels of IL-10, IL-13, Eotaxin TNFβ and RANTES compared to benign reactive effusions

**Fig. 1:**
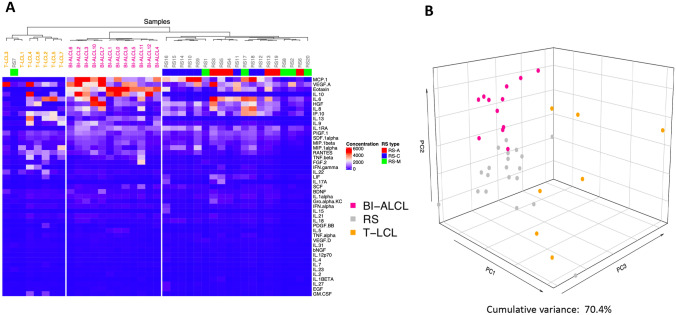
Unsupervised analyses of 45 cytokine levels in late seromas and T cell-lymphoma cell lines. Hierarchical clustering (**a**) and principal component analysis (**b**) of the levels of 45 cytokines detected in 12 breast implant-associated anaplastic large-cell lymphomas (BI-ALCL), 20 reactive seromas (RS) and 7 T-cell lymphoma cell lines (T-LCL). Reactive seromas were further subclassified as acute (RS-A, *n* = 6), mixed (RS-M, *n *= 6) and chronic-type (RS-C, *n* = 8). Cytokine profiles of BI-ALCL, RS and T-LCLB appeared clearly distinct in both unsupervised analyses

**Fig. 2:**
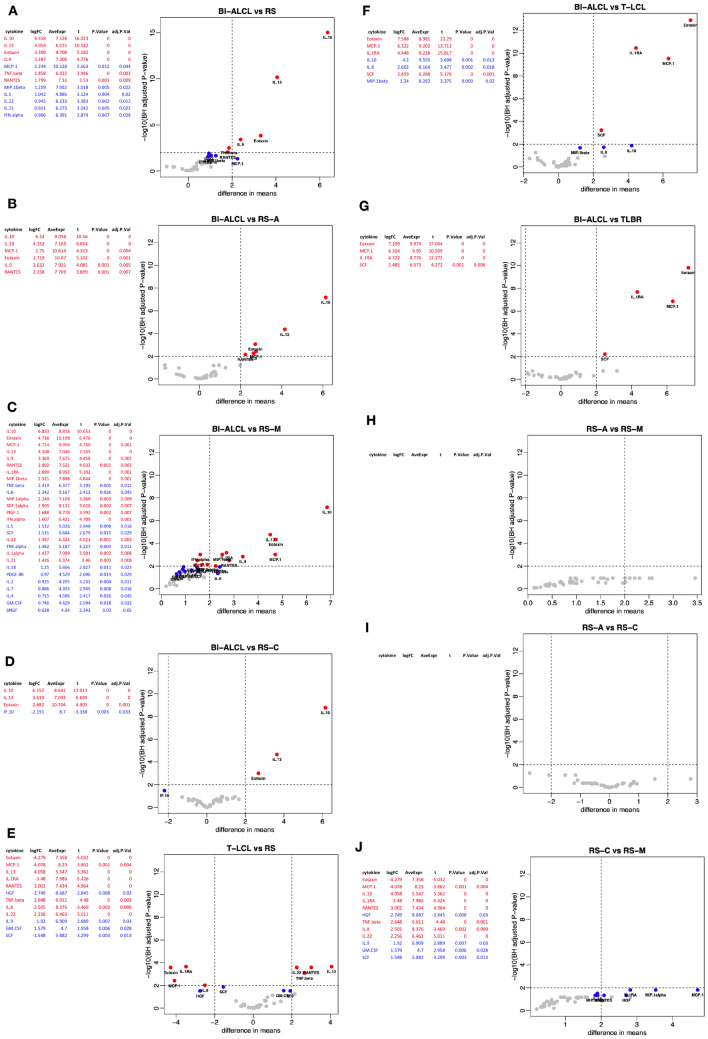
Differential analysis among different conditions of cytokine concentrations. Breast implant-associated anaplastic large-cell lymphoma (BI-ALCL) *versus* all reactive seromas (RS) (**a**). BI-ALCL *versus* acute-type reactive seromas (RS-A) (**b**). BI-ALCL *versus* mixed-type reactive seromas (RS-M) (**c**). BI-ALCL *versus* chronic-type reactive seromas (RS-C) (**d**). T-cell lymphoma cell lines (T-LCL) *versus* RS (**e**). BI-ALCL *versus* T-LCL (F). BI-ALCL *versus* BI-ALCL-derived cell line (TLBR) (**g**). RS-A *versus* RS-M (**h**) and *versus* RS-M (**i**). RS-C *versus* RS-M (**j**). For each horizontal panel significant results are listed in the table in decreasing order of log-fold change (moderated t-statistics, adjusted *p* value < .01, BH procedure) and illustrated with a volcano plot in which the vertical dashed line correspond to fourfold up and down (+ 2,  – 2 on log2 scale) change, and the horizontal dashed line represents a *p* value of 0.01 so that all cytokines above this line are deemed statistical significant with respect to that cut-off. In both, tables and volcano plots, significant cytokines with *p* value < 0.01 are indicated in red color, whereas those with *p* value < 0.05 are in blue. Grey dots represent non-significant cytokines

**Fig. 3:**
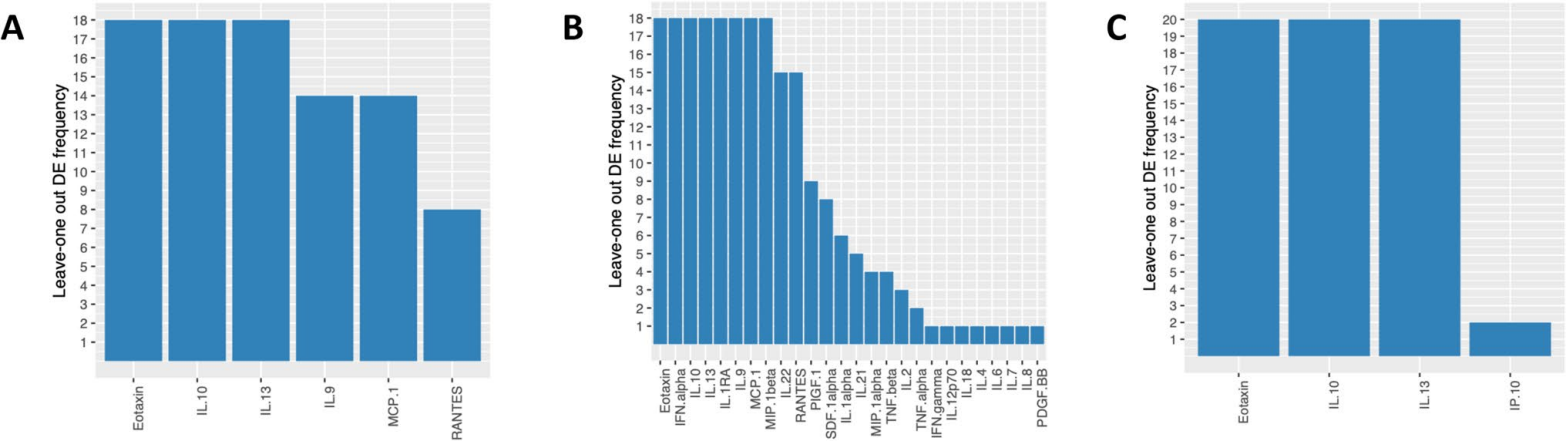
Leave-one out differential expression analysis. For each comparison BI-ALCL vs RS-A (12 BI-ALCL samples, 6 RS-A samples) (**a**); BI-ALCL vs RS-M (12 BI-ALCL samples, 6 RS-M samples) (**b**) and BI-ALCL vs RS-C (12 BI-ALCL samples, 8 RS-C samples) (**c**) the histograms report the number of times a cytokine is differentially expressed (*p* < 0.01) removing any one sample from the dataset. Only cytokines differentially expressed (*p* < 0.001) in at least one leave-one-out round are shown

We simultaneously analyzed the concentrations of 45 different cytokines, chemokines and growth factors in the supernatant of 32 late seromas, including 12 BI-ALCL and 20 benign reactive seromas (RS), and in the supernatant of 7 T-cell lymphoma cell lines (T-TCL) using a multiplex assay. Based on the cellular composition of the inflammatory infiltrate, the RS were categorized as acute-type (*n* = 6) when neutrophils represented > 50% of the total cellularity, mixed-type (*n* = 6) when composed of a variable amount of neutrophils (ranging from 5 to 50% of the total cellularity), monocytes and lymphocytes, and chronic-type (*n* = 8) when composed mainly by lymphocytes, monocytes and by sporadic polymorphonuclear granulocytes, mainly eosinophils (< 5% of the total cellularity), as previously reported [5]. First, we conducted an unsupervised clustering analysis of the cytokine levels in the different samples. Strikingly, BI-ALCL, RS, and T-TCL appeared clearly distinct by both hierarchical clustering and multidimensional scaling analysis (Fig. 1a, b). We then conducted a supervised comparison between BI-ALCL and RS which revealed as most differentially expressed cytokines (*p* < 0.001) IL-10, IL-13, Eotaxin, IL-9, TNFβ and RANTES (Fig. 2a). Among these, only IL-10, IL-13 and Eotaxin remained significantly more expressed in BI-ALCL when compared to each of the three different types of RS (acute, mixed and chronic) (Fig. 2b–d). Of note, IL-6 was not differentially represented in BI-ALCL versus RS. To assess the robustness of the genes found to be differentially expressed, we conducted a leave-one-out (LOO) analysis for each comparison (i.e. BI-ALCL versus RS-A, BI-ALCL versus RS-C, and BI-ALCL versus RS-M) by leaving out one sample each time and counting the number of times each cytokine was differentially expressed (*p* < 0.01). We considered robust only those cytokines differentially expressed in spite of the removal of any one sample in the dataset. Overall, we observe that only Eotaxin, IL-10 and IL-13 result robust in all the three comparisons (Fig. 3a–c), making them good candidates for distinguishing BI-ALCL from any types of benign seroma.

We then compared BI-ALCL to T-LCL and the most significant differentially expressed cytokines were: Eotaxin, MCP1, IL-1RA and SCF (Fig. 2f). The same result was obtained when BI-ALCL clinical samples were compared to TLBR cell lines only, which are established cell lines derived from BI-ALCL samples [9] (Fig. 2g). These results may suggest that in BI-ALCL seromas Eotaxin, MCP1, IL-1RA and SCF might be produced at higher levels by the neoplastic cells or, alternatively, by the non-neoplastic cells (i.e. lymphocytes and monocytes) present in the microenvironment. The latter hypothesis is further supported by the comparison of T-LCL to RS. Indeed, among the cytokines more abundant in RS, there were Eotaxin, MCP1, IL-1RA, and SCF whereas among those produced by T-LCL, there were IL-13, RANTES, TNFβ, IL-22, and IL-9 (Fig. 2e). Interestingly, in contrast to what we observed for BI-ALCL, T-LCL were not characterized by higher levels of IL-10 compared to RS. This diversity could be related to the lack of an microenvironmental priming effect on tumor cells under in vitro growth conditions of stabilized lymphomatous cell lines, which should be considered as a limitation to their translational use.

No significant differences were found upon comparison of acute (RS-A) versus mixed (RS-M) or chronic (RS-C) type effusions (Fig. 2h–i). Whereas, MCP1, MIP1α, IL-1RA, HGF, RANTES, IL-9, IL-18 and MIP1β were found to be significantly more abundant (*p* < 0.05) in chronic compared to mixed-type seromas (Fig. 2j).

### IL-10, IL-13 and Eotaxin concentrations and IL-10/ IL-6 ratio are candidate biomarkers for early detection of BI-ALCL

Based on the median fluorescent intensity (NetMFI) values obtained by the multiplex analysis, the concentration of IL-10, IL-13, Eotaxin and IL-6 was calculated for each sample in undiluted samples (Table 1). Mean IL-10, IL-13 and Eotaxin levels were significantly higher in patients with BI-ALCL than in patients with reactive seromas (*p* < 0.001). In particular, in patients with BI-ALCL, the mean concentration levels were for IL-10: 2601.06 pg/mL (range 58.36–6950), for IL-13: 1403.69 pg/mL (range 68.64–4950.67), for Eotaxin: 668.49 pg/mL (range 424.98–954.65), and for IL-6: 7965.87 pg/mL (range 159.81—42,900), whereas in patients with reactive seromas, the mean concentration levels were for IL-10: 3.82 pg/mL (range 0.35–19.69), for IL-13: 12.97 pg/mL (range 0.83–67.45), for Eotaxin: 179.54 pg/mL (range 0.31–671.47), and for IL-6: 5732.29 pg/mL (range 6.12–42,900).

**Table 1:** IL-10, IL-13, Eotaxin and IL-6 measurements in late peri-implant breast seromas

	Mean ± SD (pg/mL)	Range (pg/mL)	*p* value
IL-10**IL-10**			
BI-ALCL	2601.06 ± 2284.90	58.36 – 6950	BI-ALCL vs RS-All *p* < 0.001
RS-All	3.82 ± 4.53	0.35 – 19.69	BI-ALCL vs RS-A *p* < 0.001
RS-A	3.34 ± 2.81	1.46 – 8.40	BI-ALCL vs RS-M *p* < 0.001
RS-C	4.04 ± 2.72	0.48 – 7.86	BI-ALCL vs RS-C *p* < 0.001
RS-M	4.01 ± 7.71	0.35 – 19.69	BI-ALCL vs T-LCL *p* = 0.002
T-LCL	1136,43 ± 2582,54	0.38 – 6950	T-LCL vs RS-All *p* = 0.034
**IL-6**			
BI-ALCL	7965.87 ± 12,041.76	159.81 – 42,900	BI-ALCL vs RS-All *p* < 0.001
RS-All	5732.29 ± 10,539.82	6.12 – 42,900	BI-ALCL vs RS-A *p* = 0.733
RS-A	7141.83 ± 7699.36	1353.45 – 19,149.33	BI-ALCL vs RS-M *p* = 0.022
RS-C	3378.96 ± 5746.51	6.12 – 16,904.51	BI-ALCL vs RS-C *p* = 0.059
RS-M	7460.54 ± 17,372.17	7.42 – 42,900	BI-ALCL vs T-LCL *p* = 0.435
T-LCL	14,052.88 ± 19,822,55	4.19 – 42,900	T-LCL vs RS-All *p* = 0.674
**IL-13**			
BI-ALCL	1403.69 ± 1367.37	68.64 – 4950.67	BI-ALCL vs RS-All *p* < 0.001
RS-All	12.97 ± 16.62	0.83 – 67.45	BI-ALCL vs RS-A *p* < 0.001
RS-A	5.72 ± 6.51	0.83 – 17.86	BI-ALCL vs RS-M *p* < 0.001
RS-C	23.52 ± 21.83	3.37 – 67.45	BI-ALCL vs RS-C *p* < 0.001
RS-M	6.14 ± 6.56	3.37 – 19.53	BI-ALCL vs T-LCL *p* < 0.001
T-LCL	5488.42 ± 6166.29	1.31 – 13,800.00	T-LCL vs RS-All *p* = 0.610
**Eotaxin**			
BI-ALCL	668.49 ± 154.72	424.98 – 954.65	BI-ALCL vs RS-All *p* < 0.001
RS-All	179.54 ± 216.96	0.31 – 671.47	BI-ALCL vs RS-A *p* < 0.001
RS-A	184.72 ± 239.00	20.69 – 654.35	BI-ALCL vs RS-M *p* < 0.001
RS-C	238.58 ± 252.77	17.90 – 671.47	BI-ALCL vs RS-C *p* < 0.001
RS-M	95.65 ± 135.08	0.31 – 358.65	BI-ALCL vs T-LCL *p* < 0.001
T-LCL	5.73 ± 6.55	1.36 – 19.64	T-LCL vs RS-All *p* < 0.001
**IL-10/IL-6 RATIO**			
BI-ALCL	3.04 ± 6.04	0.01 – 20.43	BI-ALCL vs RS-All *p* < 0.001
RS-All	0.02 ± 0.03	0 – 0.10	BI-ALCL vs RS-A *p* < 0.001
RS-A	0.001 ± 0.002	0 – 0.01	BI-ALCL vs RS-M *p* = 0.002
RS-C	0.03 ± 0.04	0 – 0.09	BI-ALCL vs RS-C *p* = 0.001
RS-M	0.03 ± 0.04	0.001 – 0.10	BI-ALCL vs T-LCL *p* = 0.081
T-LCL	3.29 ± 8.14	0 – 21.73	T-LCL vs RS-All *p* = 0.274

**Fig. 4:**
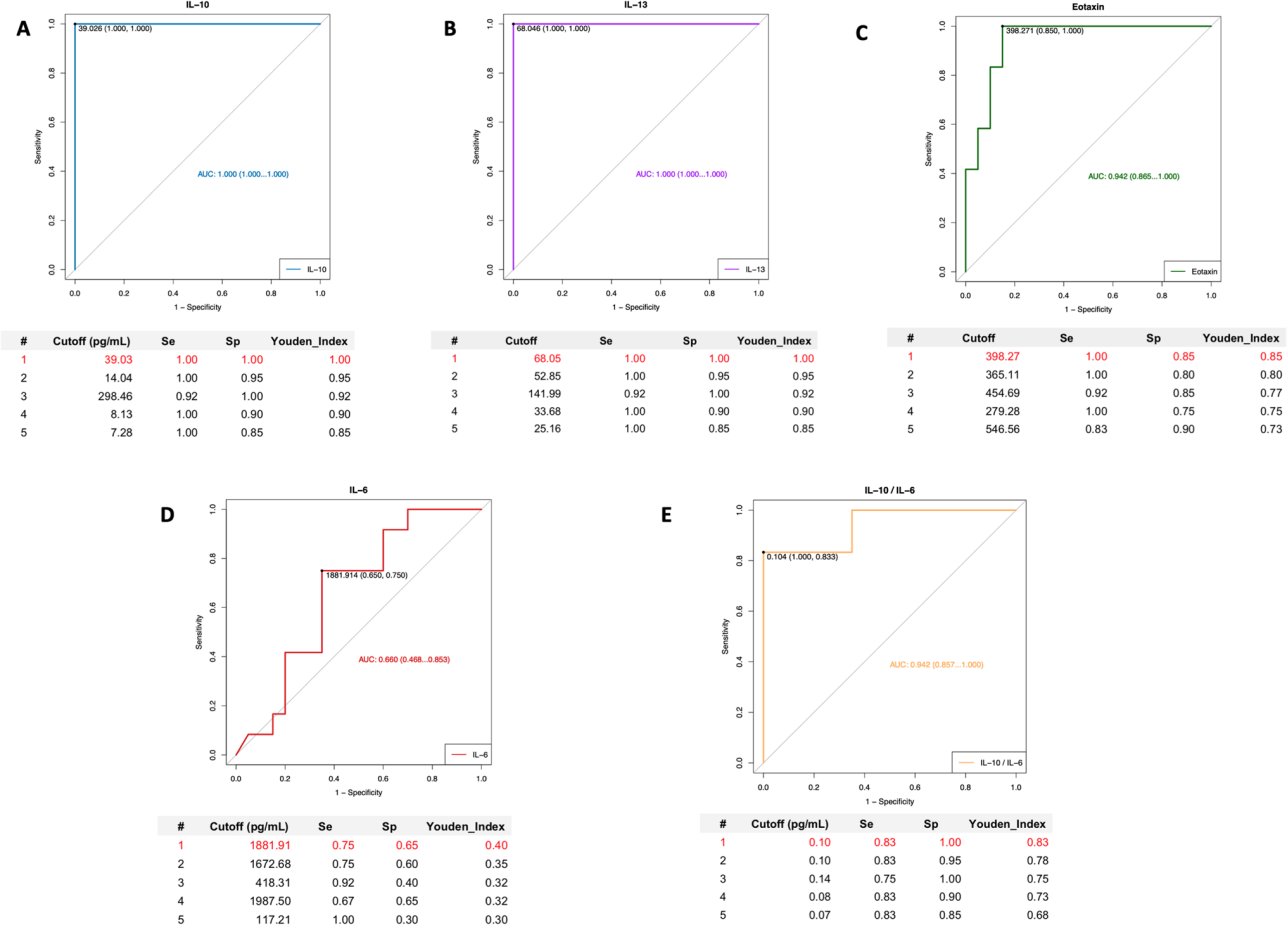
Receiver-operator characteristic (ROC) curves of IL-10, IL-13, Eotaxin and IL-6 cytokine concentrations and of IL-10/IL-6 ratio. Optimal cut-off values (Youden Index) are marked in red. IL-10: sensitivity (Se) 100%, specificity (Sp) 100% at 39.03 pg/mL (AUC. 1; 95% CI. 1.00–1.00) (**a**); IL-13: sensitivity (Se) 100%, specificity (Sp) 100% at 68.05 pg/mL (AUC. 1.00; 95% CI. 1.00–1.00) (**b**); Eotaxin: sensitivity (Se) 100%, specificity (Sp) 85% at 398.27 pg/mL (AUC. 0.94; 95% CI. 0.86–1.00) (**c**); IL-6: sensitivity (Se) 75%, specificity (Sp) 65% at 1881.91 pg/mL (AUC. 0.66; 95% CI. 0.47–0.85) (**d**); IL-10/IL-6: sensitivity (Se) 83%, specificity (Sp) 100% at 0.10 (AUC. 0.94; 95% CI. 0.86–1.00) (**e**). *AUC* area under the curve and *CI* confidence interval

We then calculated the ability of these cytokines to identify BI-ALCL among all types of seromas using a receiver operating characteristic curve (ROC) (Fig. 4a–c). ROC curves showed that a cutoff of 39.03 pg/mL for IL-10 and of 68.05 pg/mL for IL-13 was both associated with sensitivity (Se) of 100% and specificity (Sp) of 100% (Youden index = 1), whereas a cutoff of 398.27 pg/mL for Eotaxin was associated with sensitivity (Se) of 100% and specificity (Sp) of 85% (Youden index = 0.94). ROC curve for IL-6 shows the poor performance of IL-6 as a diagnostic biomarker in identifying patients with BI-ALCL (Fig. 4d).

**Fig. 5:**
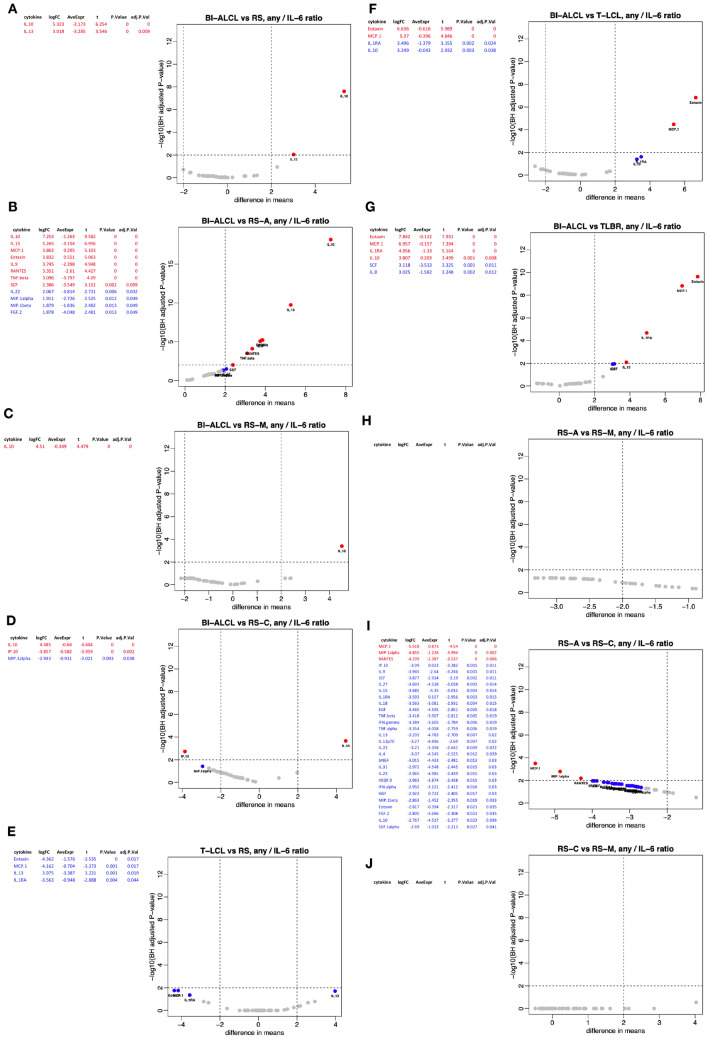
Differential analysis among different conditions of the *ratio* of each cytokine concentration to IL-6 concentration. Breast implant-associated anaplastic large-cell lymphoma (BI-ALCL) *versus* all reactive seromas (RS) (**a**). BI-ALCL *versus* acute-type reactive seromas (RS-A) (**b**). BI-ALCL *versus* mixed-type reactive seromas (RS-M) (**c**). BI-ALCL *versus* chronic-type reactive seromas (RS-C) (**d**). T-cell lymphoma cell lines (T-LCL) *versus* RS (**e**). BI-ALCL *versus* T-LCL (**f**). BI-ALCL *versus* BI-ALCL-derived cell line (TLBR) (**g**). RS-A *versus* RS-M (**h**) and *versus* RS-M (**i**). RS-C *versus* RS-M (**j**). For each horizontal panel significant results are listed in the table in decreasing order of log-fold change (moderated t-statistics, adjusted *p* value < .01, BH procedure) and illustrated with a volcano plot in which the vertical dashed line correspond to fourfold up and down (+ 2,  – 2 on log2 scale) change, and the horizontal dashed line represents a *p* value of 0.01, such that all cytokines above this line are deemed statistical significant with respect to that cut-off. In both, tables and volcano plots, significant cytokines with *p* value < 0.01 are indicated in red color, whereas those with *p* value < 0.05 are in blue. Grey dots represent non-significant cytokines

Based on these results, both IL-10 and IL-13 concentrations appeared as the best diagnostic biomarkers for BI-ALCL. However, since the same cytokine measurement could be affected by different techniques not calibrated to the same international standards [18], we tested the performance of an approach based on the adoption of ratios among paired cytokine levels. Interestingly, the IL-10-to-IL-6 ratio was among the most significant (logFC > 4; *p* < 0.001) (Supplementary Table 1). This result was of particular relevance since the IL-10/IL-6 ratio is currently being used for the diagnosis of another effusion-lymphoma e.g. the diffuse B-cell lymphoma of the vitreous [18]. In addition, IL-6 was considered a good denominator because it is a pro-inflammatory cytokine detectable in all seromas [8]. We then repeated the differential analysis looking for any cytokine produced in comparison to IL-6 in the different conditions (Fig. 5a–j) and, again IL-10 stood out as the most significant one in BI-ALCL *versus* RS (Fig. 5a). This was further confirmed when BI-ALCL was compared to the three different types of RS (Fig. 5b–d). In contrast, the IL-10-to-IL-6 ratio did not appear significant in the comparison of T-LCL to RS or to BI-ALCL (Fig. 5e, f).

**Fig. 6:**
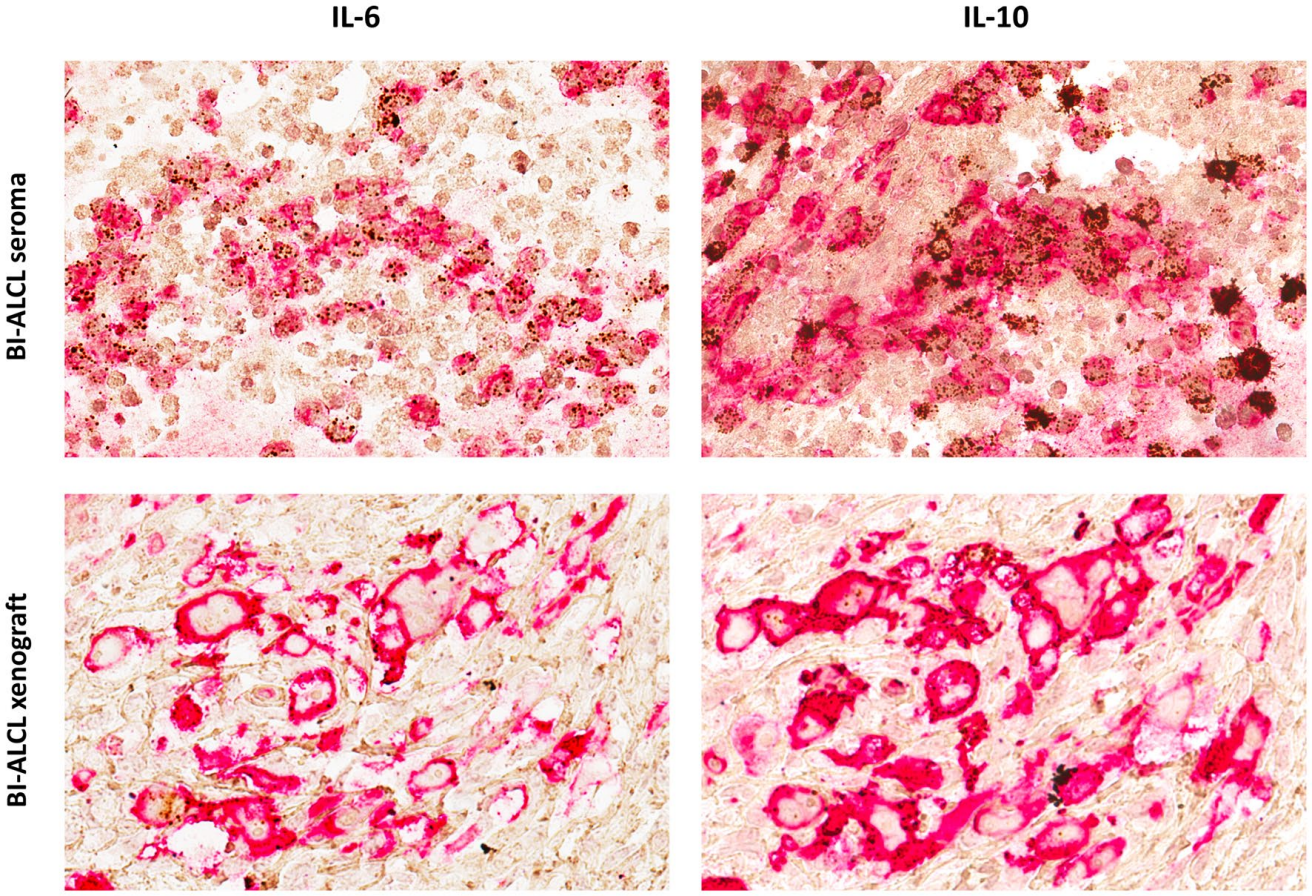
IL-10 and IL-6 mRNA in CD30-positive BI-ALCL tumor cells. Immunohistochemistry for CD30 (red staining) and in situ hybridization for IL-6 and IL-10 mRNA (brown dots) in a BI-ALCL seroma (upper panel) and in a BI-ALCL xenografted onto an NGS mouse (lower panel) (original magnification × 400). CD30-positive tumor cells showed both IL-6 and IL-10 mRNA expression. In the neoplastic effusion the brown dots for IL-10 appeared more abundant than those for IL-6

We then evaluated the potential of the IL-10/IL-6 ratio to identify BI-ALCL samples. In our sample cohort, the mean level of IL-10/IL-6 was 3.04 (range 0.01–20.43) in BI-ALCL and 0.02 (range 0–0.10) in reactive seromas (Table 1). The ROC curve (Fig. 4e) showed that IL-10-to-IL-6 ratio higher than 0.104 was associated with a Se of 83% and a Sp of 100% (Youden index = 0.83). Because BI-ALCL are composed of a large number of tumor cells associated with a small proportion of reactive cells including macrophages, small lymphocytes and eosinophils, to further confirm the production of IL-10 and IL-6 by the BI-ALCL tumor cells, we performed in situ hybridization (ISH) for IL-10 and IL-6 mRNA and IHC for CD30 on paraffin cell blocks of a BI-ALCL seroma and of a primary cultured BI-ALCL xenografted onto an NGS mouse (Fig. 6). Hybridization signals (brown dots) for IL-10 or IL-6 were found in the cytoplasm of CD30-positive cells in both the BI-ALCL seroma and in the BI-ALCL xenograft, which consisted of pleomorphic tumor cells with highly atypical nuclei and prominent nucleoli encircling central necrotic areas (Fig. 6). Of note, a higher number of signals for IL-10 were detected, as compared with IL-6 ones, especially in BI-ALCL seromas.

**Fig. 7:**
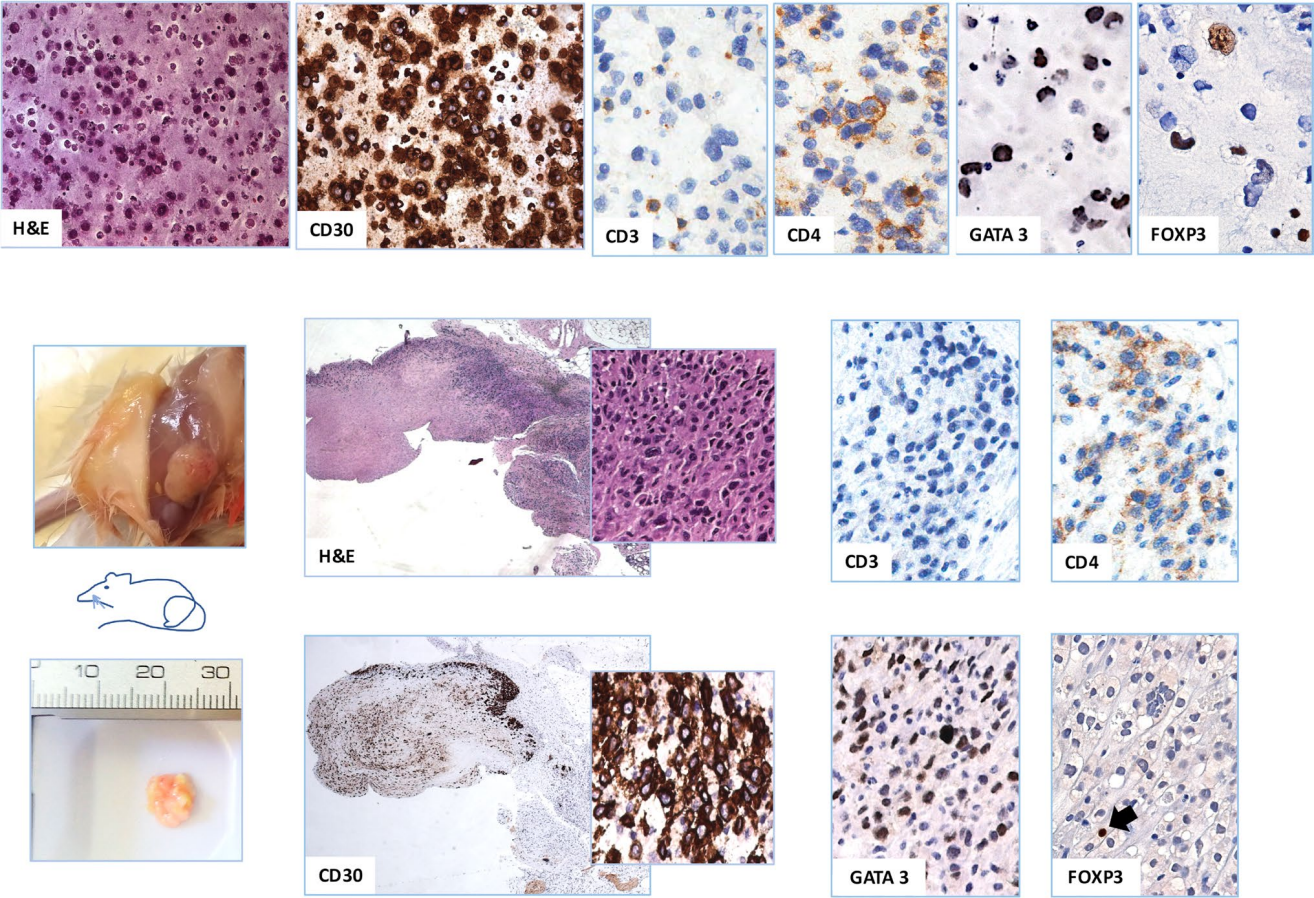
GATA3 and FoxP3 expression in BI-ALCL. Representative images of CD30, CD3, CD4, GATA3 and FoxP3 immunohistochemical expression in tumor cells of BI-ALCL seroma (upper panel; original magnification H&E and CD30 × 200, CD3, CD4, GATA3, FoxP3 × 400) and of the same BI-ALCL xenografted onto an NGS mouse (lower panel; macroscopy and histology of the tumor mass; original magnification H&E and CD30 × 25, inserts × 200, CD3, CD4, GATA3, FOXP3 × 400). In both the seroma and xenograft there was expression of CD4, GATA3 and FOXP3 in a proportion of CD30-positive tumor cells with a fewer number of FoxP3 + cells compared to those expressing GATA3. CD3 was negative in both samples

The peculiar IL-10/IL-6 imbalance within the high levels of IL-10, IL-13 and Eotaxin of BI-ALCL represent a clue to the BI-ALCL putative cell of origin. Previously, we have observed the expression of the Th2-associated transcription factor GATA3 and of the T-regulatory cell-associated marker FOXP3 in a proportion of BI-ALCL samples [19, 20]. To support the assumption that BI-ALCL xenograft retained the immunophenotype of BI-ALCL seroma upon adaptation to the host environment, we immunostained the seroma and the related xenograft for CD30, CD3, CD4, GATA3 and FOXP3 (Fig. 7), which revealed expression of CD4, GATA3 and FOXP3 in a proportion of CD30-positive tumor cells in both the samples with a lower number of FOXP3 + cells compared to GATA3 + cells.

## Discussion

The precise pathobiology of BI-ALCL remains poorly understood. Chronic inflammation associated with stable activation of the JAK-STAT3 pathway has been extensively proposed as the possible mechanism that underlies proliferation and transformation of the T cells [21–25]. In this view, cytokine profiling may help in providing insight into the cell of origin of BI-ALCL, also proving of diagnostic utility. Cytokines and chemokines are critical mediators of communication for the immune system. The cytokine pattern that is released from one cell depends primarily on the nature of the antigenic stimulus and on the functional commitment of the cell being stimulated [26]. Using a multiplex protein assay, we demonstrated that BI-ALCL has a distinctly different cytokine profile when compared with different types of benign seromas characterized by high levels of IL-10, IL13, Eotaxin, IL9, TNFβ and RANTES.

IL-13 and IL-9 are Th2 cell-associated cytokines [27]. In particular, IL-13 plays a key role in asthma, allergy, fibrosis and other pathological processes sustained by eosinophils [28, 29] whereas IL-9, initially described as T cell growth factor, is a pleiotropic cytokine linked to tumor immunity, immunity to pathogens, allergy, and autoimmune disease [30]. In addition to Th9 cells, Th2 and Th17 cells as well as induced Foxp3 + regulatory T cells (iTregs) cells also produce IL-9 [31, 32]. Of note, IL-9 has been demonstrated to be an autocrine growth factor in systemic ALK + ALCL [33]. Furthermore, IL-9 secreted by Hodgkin’s Reed Sternberg cells has been implicated in the recruitment of eosinophils in the classical Hodgkin Lymphoma microenvironment [34].

Eotaxin-1 (CCL11) and RANTES (CCL5) are also implicated in the recruitment of eosinophils into inflammatory sites during allergic reactions [35, 36]. Our data show high levels of Eotaxin in BI-ALCL but not in T-cell lymphoma cell lines supporting the hypothesis that it is secreted by normal cells of the microenvironment, activated by tumor cells. Similarly, cHL cells do not express eotaxin but produce IL-13 and TNF-α which induce eotaxin expression in co-cultured dermal fibroblasts [37]. Although eosinophils are not a constant feature, they are variably present in BI-ALCL (Supplementary Fig. 1) [19, 38].

TNF-β, also known as lymphotoxin alpha (LTα), in addition to promoting lymphoid tissue development, contributes to the effector responses of both the innate and the adaptive immune systems. Although data indicate that LT might be essential for Th2 cell development, it is traditionally regarded as a hallmark cytokine in Th1 cell responses [39, 40].

IL-10 may participate in mediating and/or regulating the functions of Th2 cells [41], but it is mainly produced by Tregs (i.e. thymus-derived Tregs and induced Tregs) to quench pro-inflammatory responses of both innate and adaptive immune cells preventing excessive tissue damage caused by bacterial and viral infections [42]. From our previous gene expression profiling data, a higher level of IL-10 mRNA emerged in BI-ALCL as compared to normal CD4 + T cells [20]. Herein we further demonstrated that the production of IL-10 in BI-ALCL is predominantly intrinsic to CD30-positive tumor cells, as shown by in situ hybridization data on seroma and xenograft.

Altogether, the abundance of IL-10, IL-13, IL-9, Eotaxin, Rantes and TNF-α and the frequent expression of GATA3 and FoxP3 transcription factors by neoplastic cells suggest that in BI-ALCL, a skewing towards Th2 and Treg cells occurs. We may postulate that in BI-ALCL pathogenesis, the implant may have elicited a Th2-type response with accumulation of T cells, mast cells, monocytes and eosinophils and that, also consequently to the activation of the STAT3 pathway, IL-10-producing Tregs may eventually be recruited or induced as a feedback mechanism. We have previously shown that gene expression profiling and immunohistochemical data suggest either activation-induced FoxP3 expression or a T helper-like regulatory T-cell status in a proportion of BI-ALCL with the upregulation of RORC, IL17A genes and of FoxP3 protein (20). Several reports also suggest that there is plasticity between Th2 cells and iTregs [43], which would be in keeping with our findings. In addition, evidence suggests that Tregs in the periphery can become unstable and dampen Foxp3 expression, adopting some effector functions but still producing high amounts of IL-10 [44].

One important element of this study consists in the comparison of the BI-ALCL-associated cytokine milieu with that of the three different types of reactive effusions (i.e. acute, chronic and mixed), which revealed that high levels of IL-10, IL-13 and Eotaxin are able to differentiate BI-ALCL from all types of benign seromas. Moreover, IL-10/IL-6 ratio higher than 0.1 identified 10 out of 12 BI-ALCL, which means that 83 out of 100 seromas would be correctly classify as BI-ALCL. These findings are similar to what observed in primary central nervous system lymphoma (PCNSL) and in primary vitreoretinal lymphoma (PVRL, a subtype of PCNSL), in which the uses of IL-10 and IL-10/IL-6 ratio measurements have been proposed as diagnostic tools. Indeed, in PCNSL and PVRL, increased IL-10 levels in the vitreous or cerebrospinal fluids have been demonstrated [45–48]. Furthermore, the detection of an IL-10-to-IL-6 ratio greater than 1 in the vitreous and greater than 0.72 in the cerebrospinal fluid is considered useful for differentiating between PVRL and intraocular infectious diseases, and PCNL from intracranial infections, respectively [18, 48–52]. In the case of PVRL, this led to the suggestion for screening of suspected uveitis cases by evaluating IL-10 values and IL-10/IL-6 ratio before vitreous biopsy [53]. On the basis of our findings, IL-10 and IL-6 levels analysis may also be envisaged for late peri-implant seromas of the breast on the fluid undergoing cytological examination and microbiological culture. Indeed, cytokine measurement is a reliable and easy test that does not require additional special handling of the aspirated fluid or particular expertise in reading results. This approach, if appropriately validated in ad-hoc designed prospective studies with a cost-effective test, might represent a new diagnostic tool to support cytology in the screening of late seromas. In addition, the inclusion of even more rare breast implant-associated Epstein–Barr virus (EBV)-positive large B-cell lymphomas [54] would test the ability of the identified BI-ALCL-associated cytokine profile and of the IL-10/IL-6 value to discriminate between BI-ALCL and the EBV + large B-cell lymphomas.

In conclusion, our results show that BI-ALCL has a distinctive cytokine profile, mainly characterized by high levels of IL-10, IL-13, Eotaxin and IL-10/IL-6 ratio, which might represent additional biomarkers to be used in daily clinical practice for screening late seromas.

## Electronic supplementary material

Below is the link to the electronic supplementary material.

Supplementary file1 (XLSX 172 KB)

Supplementary file2 (TIF 35703 KB)

## Data Availability

Luminex data can be found in a data supplement available with the online version of this article.

## Abbreviations

BDNF: Brain-derived neurotrophic factor
BI-ALCL: Breast implant-associated anaplastic large-cell lymphoma
CCL5 (RANTES): Chemokine C–C motif ligand 5
EGF: Epidermal growth factor
FGF2: Fibroblast growth factor
GM-CSF: Granulocyte–macrophage colony stimulating factor
GRO-α: Growth-related oncogene protein-α
HGF: Hepatocyte growth factor
IFN-α, IFN-γ: Interferon-α, -γ
IL-10, IL-6: Interleukin 10, -6
IL-1RA: Interleukin 1 receptor antagonist
IP-10, CXCL10: IFN-γ-inducible protein 10
LIF: Leukemia inhibitory factor
MCP-1: Monocyte chemotactic protein 1
MIP-1β (CCL4), MIP-1α: Macrophage inflammatory protein type 1β, 1α
PIGF-1: Placenta growth factor 1
RS: Reactive seromas
SCF: Stem cell factor
SDF-1α: Stromal cell-derived factor 1
TNF-α, TNF-β: Tumor necrosis factor-α
VEGF-A, VEGF-D: Vascular endothelial growth factor-A, -D
