# Supplementary figures and images for: IL-10, IL-13, Eotaxin and IL-10/IL-6 ratio distinguish breast implant-associated anaplastic large-cell lymphoma from all types of benign late seromas
Source: Cancer Immunol Immunother. 2020 Nov 4;70(5):1379–92. doi: 10.1007/s00262-020-02778-3 (PMC8053183; doi:10.1007/s00262-020-02778-3)

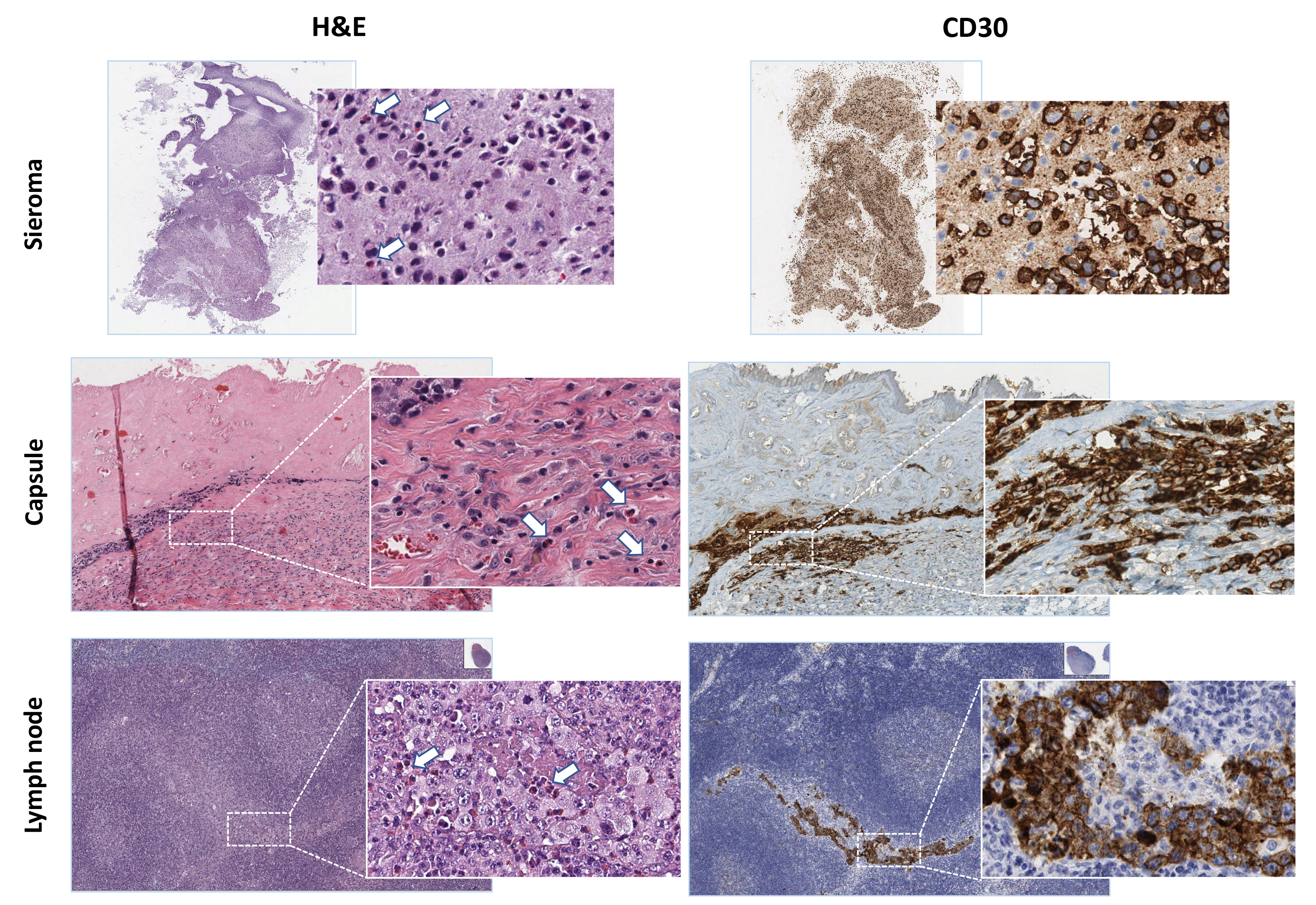

Supplement: Supplementary file 2 — Supplementary file2 (TIF 35703 KB) [file 262_2020_2778_MOESM2_ESM.tif]
